# Examining the Applicability of Surgical Coaching Rules for Resident Autonomy in Non-teaching Hospitals

**DOI:** 10.7759/cureus.53239

**Published:** 2024-01-30

**Authors:** Amro Elhadidi, Samira Abdel Raouf, Hamdi Salama, Amged Fadl, Mohamed Abdelhalim

**Affiliations:** 1 Surgery, Mansoura University, Mansoura, EGY; 2 Surgery, Al-Azhar University, Cairo, EGY

**Keywords:** non-teaching hospitals, resident autonomy, surgical coaching, learning curve, retrospective studies

## Abstract

Introduction: This retrospective study aims to analyze the impact of standardized rules for teaching in university hospitals on surgical resident autonomy and patient safety, as measured by patient outcomes, and also examines the learning curves for residents and their impact on patient outcomes in a non-teaching hospital.

Methods: The data for the study was collected retrospectively from medical records of 2000 adult patients who went through surgical procedures from January 2020 to December 2022. Participants were categorized into two groups based on the supervision level provided by attending surgeons and residents. Appropriate statistical methods were used to analyze the data.

Results: It was observed that operative times of cases handled by both attending and resident surgeons were less than those handled by residents alone. On the other hand, the former group had a significantly higher burden of comorbidities and higher rate of perioperative complications than the latter. These results have important implications for the training of medical residents and the overall delivery of healthcare services in university hospitals.

Conclusion: The findings will also help towards better understanding of the effectiveness of these rules and their potential for improving the quality of care provided by residents in these settings.

## Introduction

Resident surgical autonomy refers to the level of independence that a surgical resident has in performing procedures and making medical decisions without direct supervision from an attending surgeon [1]. The level of autonomy can vary depending on the resident's level of experience, the complexity of the procedure, and the policies of the training program or institution. In general, surgical residency programs aim to gradually increase the level of autonomy of their residents over time [2]. During the early years of training, residents may observe and assist attending surgeons with procedures, but may not have direct involvement in the surgical process. As they gain experience and knowledge, they may be allowed to perform simpler procedures under direct supervision. Eventually, they are given more independence to perform more complex procedures on their own, with attending surgeons only present in case of emergencies [3]. The level of autonomy given to residents can be influenced by several factors, such as the number of cases they have successfully completed, their level of competence and confidence, the complexity of the procedure, and the presence of appropriate support staff and resources [4].

It is important for residents to understand their level of autonomy and to communicate openly with their attending surgeons to ensure that patient safety is maintained at all times.

Recently, training during surgery residency has undergone significant changes, primarily due to external regulatory procedures, increased emphasis on resident supervision, and changing trends in general surgery practice [5]. In the surgical community, confidence is a vital quality for surgeons. Several surveys have been conducted to assess the confidence of general surgery residents and emerging graduates. Some reports have shown that they lack confidence in performing general surgical procedures independently [6].

Surgical resident training can vary depending on the institution where the residency program is located. In some cases, residents may have the opportunity to train in private hospitals or remote locations. Private hospitals may offer unique learning opportunities, such as exposure to a different patient population, specialized surgical procedures, or access to advanced technology [7]. However, private hospitals may also have fewer resources and support systems for residents compared to larger academic medical centers. Likewise, remote hospitals may also offer unique training experiences, such as exposure to rural medicine and emergency medical situations [8]. However, remote hospitals may have limited resources and limited access to specialists, which can limit the scope of training opportunities.

University hospitals follow established faculty teaching rules and study curriculums to impart proper training to surgical residents as per the Accreditation Council for Graduate Medical Education (ACGME) guidelines. Private community hospitals offer fellowships for surgeons outside the ACGME system [7]. Lack of academic professionals during training is a huge hurdle to resident confidence, decision-making capability and autonomy [9]. Furthermore, most private and remote hospitals are shifting towards a consultant-based service which leads to limited time for trainee education. The requirement for consultants' own professional development further reduces the time available for trainees, particularly in acute specialties where traditional lecture/tutorial approaches are not feasible due to the introduction of shift systems [10]. Despite such hurdles, it is important for surgical residents to receive proper supervision and support during their training, regardless of the location of the hospital. Residents should also have access to opportunities for continuing education and professional development to ensure they are well-prepared to provide high-quality patient care after completing their residency program.

This retrospective study focuses on the implementation of standardized rules for teaching in university hospitals and examines their impact on resident autonomy and patient safety, as measured by patient outcomes. The results are useful in gaining a better understanding of the effectiveness of these rules and their potential for improving the quality of care provided by residents in these settings. These findings are crucial for the training of medical residents and the overall delivery of healthcare services in university hospitals.

The study's objective is to determine whether the same standards used in teaching and university hospitals can be applied to private and non-teaching hospitals; this is a consultant-based effort without any teaching implications.

## Materials and methods

The study's objective is to determine whether the same standards used in teaching and university hospitals can be applied to private and non-teaching hospitals; this is a consultant-based effort without any teaching implications.

The study was conducted on five residents over a three-year period (January 2020-December 2022) in a 130-bed single private hospital in Saudi Arabia (Hayat National Hospital, Al Qassim). The residents represented the core of the general surgical department. The five residents arrived at work with varying levels of expertise and hold licenses from the Saudi Commission for Health Specialists (SCFHS). The residents' different levels of surgical training (which was mostly based on operating room assistance) were observed, and a research plan for resident training that is similar to university teaching hospitals that assess residents' autonomy was adopted. The study included approximately 2000 adult patients who underwent surgical procedures during the study period. Residents' details are described in Table 1.

**Table 1:** Resident’s training and experience details

Resident gender	
Male	4
Female	1
Years of experience before commencing the study	
Years	1-3
Level of previous training	
Teaching hospital	2
Non-teaching hospital	3
On call days/month	6
Operation list/week (excluding emergency cases)	2
Work hours/day	8

The level of supervision provided by attending surgeons was used to categorize the procedures, with the cumulative experience in general and laparoscopic surgery being considered. The Attending-Resident (AR) group included cases handled by attending surgeons with resident assistance, whereas the Resident-Attendant (RA) group included cases handled by resident surgeons with attending assistance. Attending assistance in the Resident-Attendant (RA) group could be those scrubbing at operation room, onsite or on call, all collectively for simple measuring.

The data for the study was collected retrospectively from hospital medical records, which included baseline demographics viz. gender, age, body mass index (BMI), ASA scores and comorbidities, operative variables, and perioperative outcomes. Exclusion criteria for the study were cases such as exploration laparotomy, cancer patients, and major trauma patients, which were typically managed by attending physicians. Categorial data were analysed using Fisher’s exact test and continuous variables were compared using ANOVA or Kruskal Wallis test as appropriate. Statistical calculations were done using the MedCalc statistical software.

## Results

Case description of AR and RA groups

A total of 2000 cases were considered for this study. Of these, 1569 cases were attended by the AR group, while 431 cases were primarily attended by residents in the RA group. In the AR group, the most common surgeries performed were anorectal procedures and laparoscopic cholecystectomy, which accounted for 25.5% of all cases each, followed by laparoscopic appendectomy (17.2%). Open inguinal hernia repair was the most common type of hernia repair, accounting for 15.9% of all hernia cases, followed by open umbilical hernia repair (7.6%) and open ventral/incisional hernia repair (5.1%). In the RA group, the most common procedures were laparoscopic cholecystectomy (27.8%), anorectal procedures (19.7%), and open inguinal hernia repair (18.7%). Laparoscopic appendectomy was performed in 14.5% of these cases, followed by open appendectomy (5.4%) and open umbilical hernia repair (4.7%). Perforated duodenal ulcer was a rare procedure, with laparoscopic and open procedures accounting for only 0.3% and 0.5% of cases in the AR and RA groups, respectively. The case descriptions have been summarized in Table 2.

**Table 2:** Summary of cases handled by the AR and RA groups

Case Description	Attending-Resident (AR) (1569 cases)	Resident-Attendant (RA) (431 cases)
Hernia:		
Open Inguinal Hernia	250 (15.9%)	80 (18.7%)
Open Umbilical Hernia	120 (7.6%)	35 (8%)
Open Ventral/Incisional Hernia	80 (5.1%)	20 (4.7%)
Cholecystectomy:		
Laparoscopic	400 (25.5%)	120 (27.8%)
Open	12 (0.8%)	3 (0.7%)
Appendectomy:		
Laparoscopic	270 (17.2%)	63 (14.5%)
Open	30 (1.9%)	23 (5.4%)
Perforated Duodenal Ulcer:		
Laparoscopic	5 (0.3%)	0 (0%)
Open	2 (0.2%)	2 (0.5%)
Anorectal Procedures	400 (25.5%)	85 (19.7%)

Demographic details of the patients

Of the total patients operated by the AR group, 77% were male, with an average age of 56 years and an average BMI of 28. The majority of patients were physically fit and had an ASA score of 1 (61.5%), followed by an ASA score of 2 (20.5%) and 3 (18%). In the RA group, 66.8% of patients were male with an average age of 53 years and average BMI of 27. About 72.4% of patients were healthy and had an ASA score of 1.

When comparing the two groups, it was found that there were significant differences in comorbidities (p<0.001). A total of 30.5% of patients in the AR group and 19.5% in the RA group were reported to have comorbidities. These results suggest that patients primarily attended by the AR group had a higher burden of comorbidities compared to those attended the RA group. This difference may be attributed to differences in patient selection or management between the two groups. The demographic details and case quality have been summarized in Table 3.

**Table 3:** Demographic details and health conditions of the patients operated by the AR and RA groups

Variable	Attending-Resident (AR)	Resident-Attendant (RA)	p value
Male	1209 (77%)	288 (66.8%)	
Age (Years)	56 (±21%)	53 (±17%)	
BMI	28 (±8%)	27 (±9%)	
ASA Score:			
1	965 (61.5%)	312 (72.4%)	
2	322 (20.5%)	65 (15.1%)	
3	282 (18%)	54 (12.5%)	
Comorbidities	473 (30.5%)	84 (19.5%)	<0.001

Operative times

Comparing the operative times for the two groups, it was observed that the mean operative times for most procedures were higher in the RA group. The mean operative time for open umbilical hernia was longer for the AR group whereas that for laparoscopic cholecystectomy was similar for both groups. The actual operative times of different procedures by the two groups are summarized in Table 4.

**Table 4:** Operative times for the AR and RA groups for different procedures

Case description	Operative time (Minutes)	
	Attending-Resident (AR)	Resident-Attendant (RA)
Hernia:		
Open Inguinal Hernia	66 (±16)	73 (±15)
Open Umbilical Hernia	47 (±20)	45 (±26)
Open Ventral/Incisional Hernia	83 (±25)	90 (±18)
Cholecystectomy:		
Laparoscopic	48 (±19)	48 (±21)
Open	66 (35)	90 (±18)
Appendectomy:		
Laparoscopic	40 (±13)	45 (±17)
Open	35 (±20)	37 (±16)
Anorectal Procedures	35 (±20)	40 (±23)

Perioperative outcomes

The 30-day mortality rate in the AR group was 0.5% whereas there was no mortality in the RA group (p<0.001). The incidence of secondary operation including operative bleeding within 48 hours was five (0.3%) in the AR group while no such incidence was observed in the RA group. Likewise, the occurrence of venous thromboembolic events was found to be two (0.01%) and wound complications were observed in 33 (2.2%) patients operated by the AR group. Among the wound complications, 26 (1.7%) patients had a superficial infection of the surgical site and seven (0.5%) had a deep infection. In the RA group, only two (0.5%) patients operated by the RA group had superficial infection and no deep infection was observed in any patients. Statistical analysis revealed significant differences between groups with respect to all outcomes measured (p<0.001). The findings have been summarized in Table 5 and suggest that patients in the AR group experienced a greater rate of complications compared to those in the RA group.

**Table 5:** Perioperative outcomes of the cases in the two groups

Outcomes	Attending-Resident (AR)	Resident-Attendant (RA)	p value
Mortality (30-days)	7 (0.5%)	0	<0.001
Secondary operation including operative bleeding (within 48 hours)	5 (0.3%)	0	<0.001
Venous Thromboembolic Events	2 (0.01%)	0	non-significant
Wound Complications:			
Superficial surgical site infection	26 (1.7%)	2 (0.5%)	<0.001
Deep surgical site infection	7 (0.5%)	0	<0.001

## Discussion

This retrospective study compared the outcomes of patients undergoing surgical procedures with both attending and resident surgeons (AR) versus primarily resident surgeons (RA) in a remote hospital. The study included 2000 cases and analyzed demographic details, operative times, and perioperative outcomes. The results showed that the AR group performed a higher number of anorectal procedures and laparoscopic cholecystectomies while the RA group performed more laparoscopic cholecystectomies. Significant differences were also observed in patient demographics, with patients in the AR group having a higher burden of comorbidities. The mean operative times for most procedures were higher in the RA group. The most significant finding was the difference in perioperative outcomes between the two groups. Patients in the AR group had a higher rate of complications, including wound complications, secondary operations, and mortality. These results suggest that the involvement of residents in surgical procedures may not necessarily lead to worse outcomes. In fact, it may even result in better outcomes, as observed among the patients in the RA group who had no mortality and a lower rate of wound complications.

A similar retrospective report by Kunac et al. [11] studied the impact of declining resident autonomy on patient outcomes and operative times. The study was conducted using the Veterans Affairs Surgical Quality Improvement Program (VASQIP) database. It included adult patients who underwent surgery from July 1, 2004, to September 30, 2019. The study found that the rate of resident autonomy in general surgery cases has reduced by two-thirds over the 15-year study period. The cases performed by residents without the assistance of any attending surgeon were performed much faster than cases performed by a resident and attending together. There was no increase in patient morbidity or mortality in the latter case. The study concluded that it was necessary to increase operative autonomy in surgical residents is needed as the depletion of resident autonomy is not reasonable. Wojcik et al. [12] explored the feasibility of a rotation that grants structured operative autonomy to chief residents in the operating room. The study assessed its effect on patient safety and its apparent educational benefits for residents. It found that resident autonomy did not negatively impact patient outcomes, and all participants strongly agreed that the rotation led to a smooth transition from residency to fellowship or independent practice. Patient safety is a huge concern regarding resident autonomy in surgical procedures. In this regard, Putnam et al. [13] studied the perceptions of patient safety among surgical residents and perioperative personnel in an academic children's hospital. Results indicate that most personnel perceive a safer working environment than the general surgery residents in all three aspects of safety culture, teamwork, and speaking up. Junior and senior residents had lower scores for all three domains, although not statistically significant. The authors suggest that for optimal surgical education on patient, a dedicated and systematic approach is the need of the hour. Flint et al. [14] demonstrated the value of resident coaching from the viewpoint of orthopaedic residents. They used a survey-based study to determine the mentoring frequency in orthopaedic residency programs and the apparent value of mentorship. Almost half of the respondents either had a mentor or were involved in a mentoring program. Almost all residents highly ranked mentorship and expected substantial help from their mentor over the course of their medical education and career. The residents reported most satisfaction with the existence of a formal program, with residents who could select their own mentor being more satisfied than those who had their mentor assigned. The study suggests that formal mentorship programs should be established and residents should be allowed to select their own mentors. It is necessary to identify factors that limit the autonomy of surgical residents in the operating room. One survey showed that attending surgeons were most likely to give residents more responsibility if they had good clinical skills and the surgeon felt confident in their ability. However, attending surgeons were also concerned about patient safety and hospital expectations. The survey also found that many faculty members felt that work-hour regulations and increased supervision requirements were barriers to resident autonomy [15].

While considering all the above factors and perceived hurdles in achieving resident autonomy in remote, limited-access hospitals, more studies are required to validate its efficiency and demonstrate its safety regarding patient outcomes. A pilot study by Wojcik et al. [16] demonstrated an effective method to increase surgical autonomy in a remote resident-run small surgery clinic. The study evaluated the safety of the clinic in a university-based surgery training program. Ten third-year residents performed 399 procedures, with no significant difference in post-procedure complications compared to attending surgeons. The rotation increased resident autonomy and was compatible for mid-level residents, providing focused learning and enhancing their operative experience. The study suggests that a resident-run clinic is a safe and efficient way to increase operative autonomy among trainees [15]. Similarly, this study demonstrates that resident autonomy can be achieved in remote hospitals provided a learning curve is applied and strictly followed under planned supervision.

Mulita et al. emphasized key technological developments in the surgical field, with a special focus on the Internet of Surgical Things (IoST). These include telesurgery and telementoring, image-guided surgery, and patient telemonitoring. The incorporation of IoST allows for remote surgical procedures as well as real-time mentorship from experienced professionals to surgeons in distant locations. Furthermore, IoST facilitates the superimposition of preoperative imaging on live surgical camera feeds, improving precision during surgeries. Additionally, it enables remote monitoring of patients through interconnected biosensors for real-time tracking of physiological parameters and alerts to healthcare providers. These advancements highlight IoST's potential to transform surgical practice by enhancing precision, enabling remote procedures and guidance, and facilitating advanced patient monitoring outside traditional healthcare settings [17]. In non-academic medical centers, the adoption of IoST has the potential to enable resident physicians by granting them access to advanced technological resources and immediate mentorship opportunities, which in turn promotes independence and skills enhancement. Moreover, incorporating IoST into patient telemonitoring can allow residents to oversee and coordinate patient care from a distance, contributing to their professional development as well as the provision of high-quality healthcare services in non-teaching hospital environments. Through harnessing IoST capabilities, non-teaching hospitals can narrow the disparity in accessing advanced surgical tools and expertise, ultimately equipping residents to provide exceptional care while advancing their surgical proficiencies [17].

Limitations of the study

It is worth noting that this study has some limitations. Firstly, the study is retrospective and observational, and therefore, a causal relationship between the involvement of residents and the observed outcomes cannot be established. Secondly, the sample size of the study is limited and the findings cannot be generalized for the entire population. Further studies with bigger sample sizes and randomized controlled trials are required to confirm the current findings.

## Conclusions

In summary, this study investigated the learning curves of residents in non-teaching hospitals and discovered that the AR group had a higher comorbidity burden than the RA group. While the RA group required longer operative times, they had lower 30-day mortality and wound complications than the AR group. The findings suggest that, with adequate supervision, resident involvement in operative cases can positively influence patient outcomes. However, the study emphasizes the potential difficulties associated with the learning curve, such as extended operative times and increased complications. Prospective studies to assess long-term effects and investigate the role of simulation training in optimizing resident learning curves are possible future research directions. Furthermore, more research is needed to determine the optimal level of supervision and support for residents in non-teaching hospitals to ensure optimal patient outcomes.
